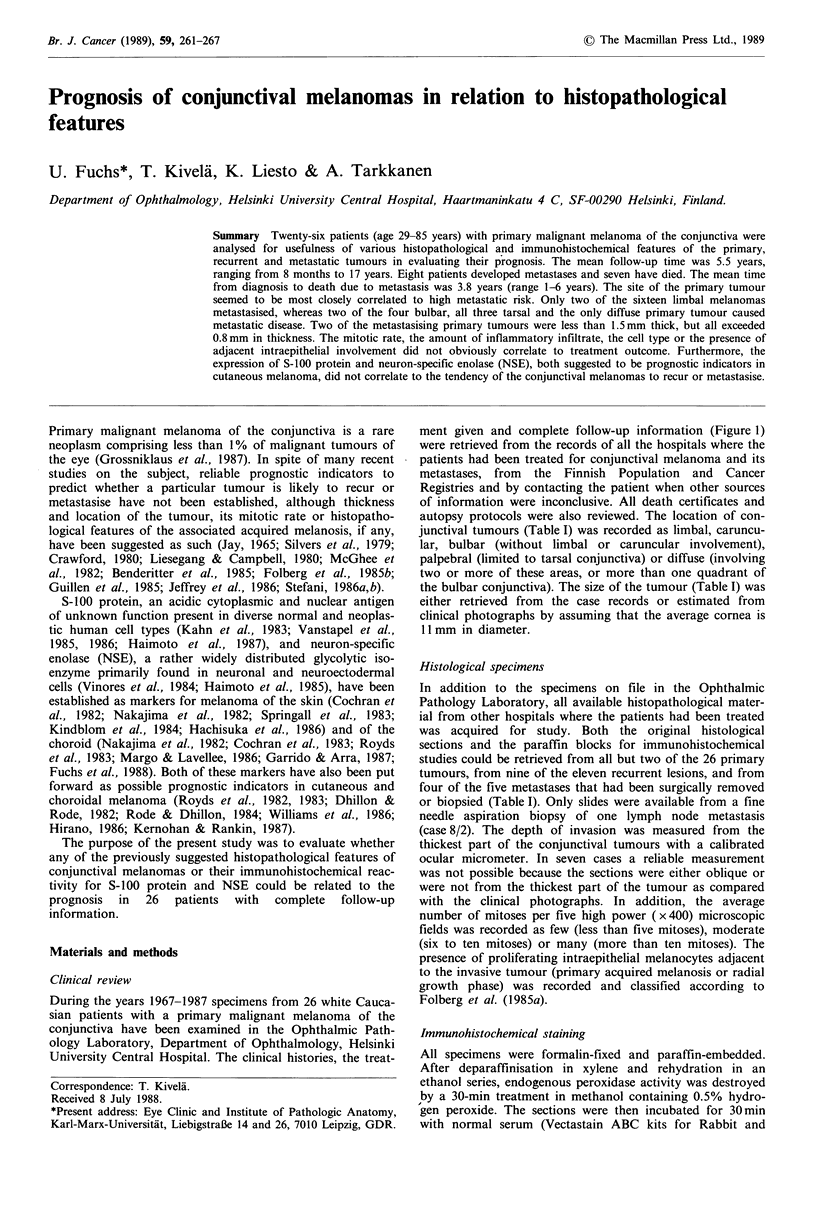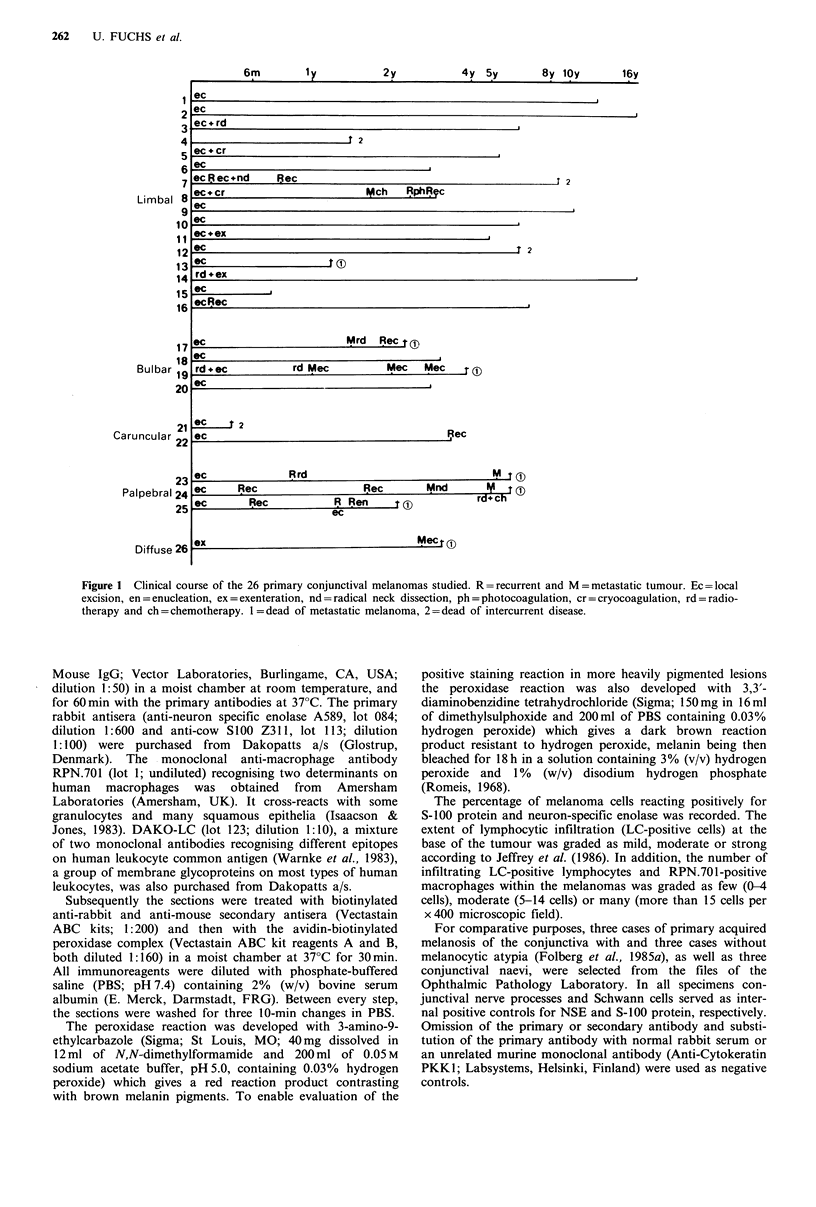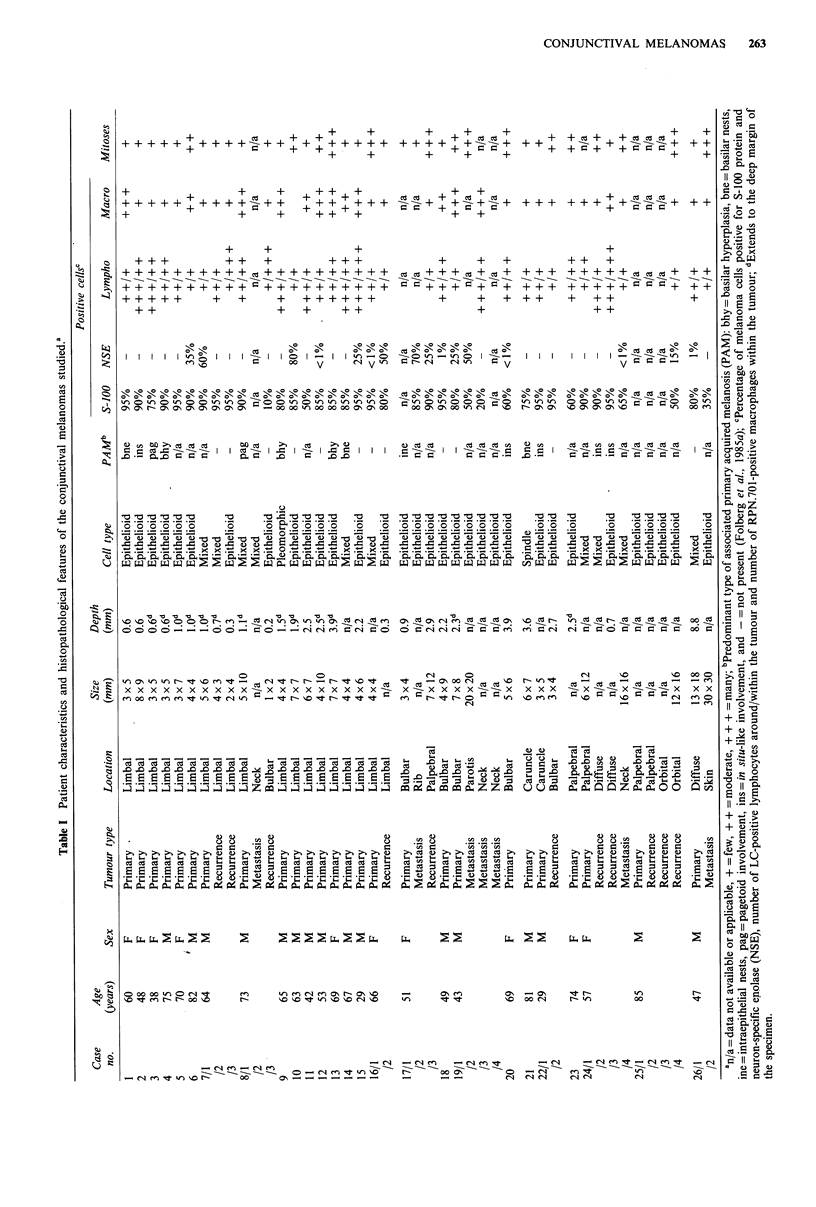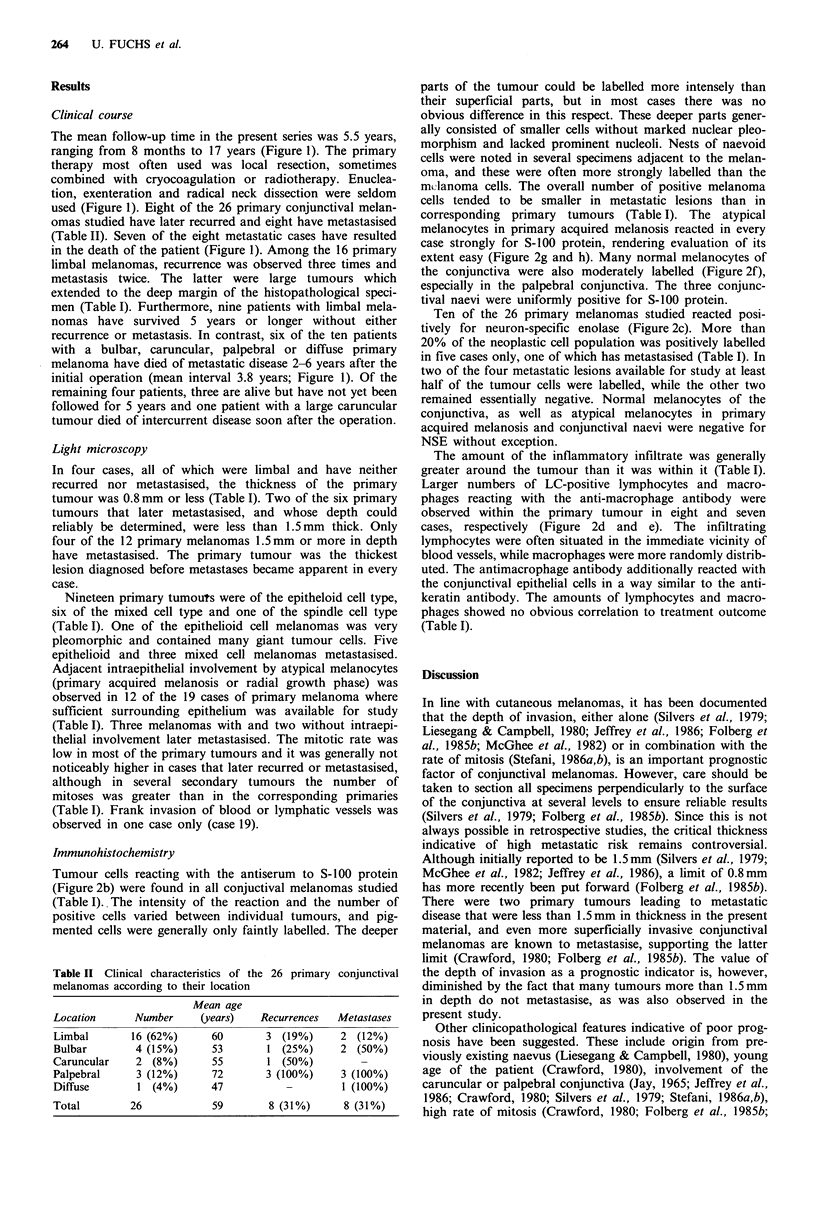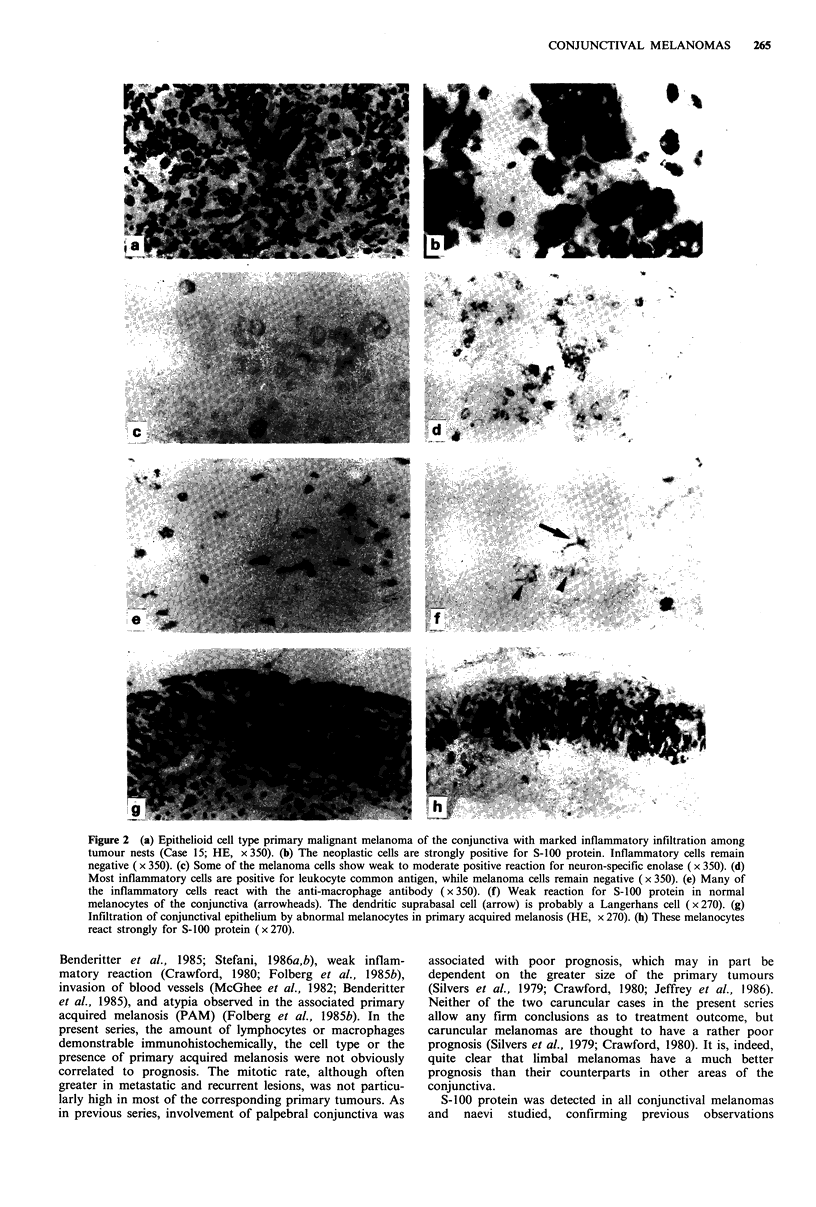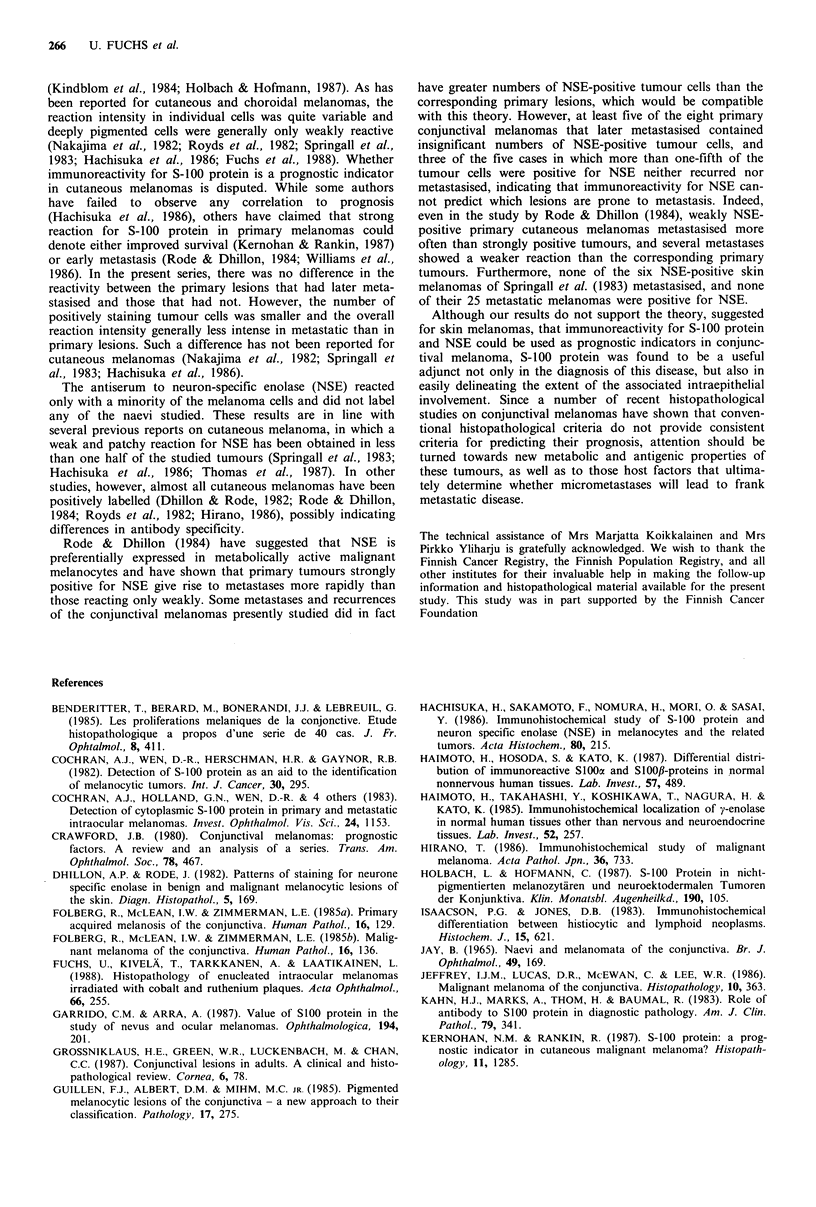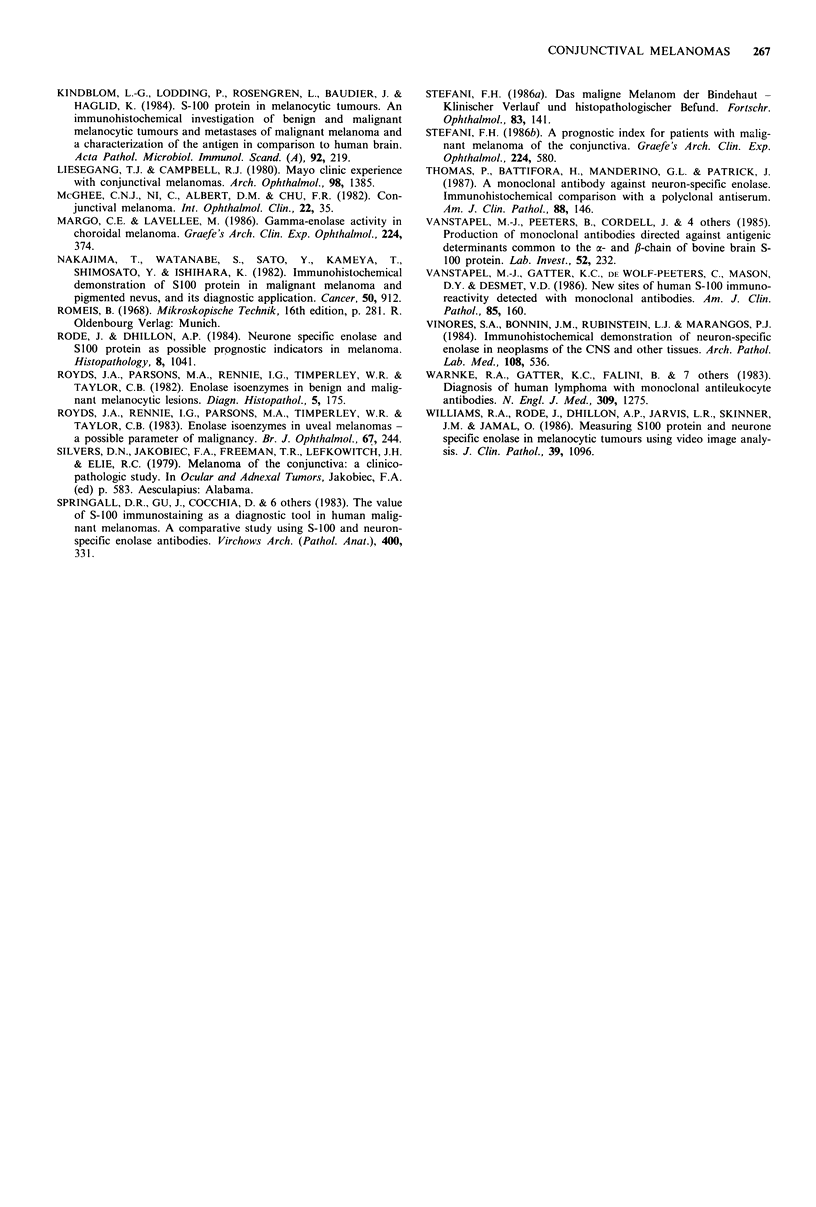# Prognosis of conjunctival melanomas in relation to histopathological features.

**DOI:** 10.1038/bjc.1989.55

**Published:** 1989-02

**Authors:** U. Fuchs, T. KivelÃ¤, K. Liesto, A. Tarkkanen

**Affiliations:** Department of Ophthalmology, Helsinki University Central Hospital, Finland.

## Abstract

**Images:**


Br. J. Cancer (1989), 59, 261-267 © The Macmillan Press Ltd., 1989

### Prognosis of conjunctival melanomas in relation to histopathological features

U. Fuchs*, T. Kivelä, K. Liesto & A. Tarkkanen

*Department of Ophthalmology, Helsinki University Central Hospital, Haartmaninkatu 4 C, SF-00290 Helsinki, Finland.*

**Summary** Twenty-six patients (age 29–85 years) with primary malignant melanoma of the conjunctiva were analysed for usefulness of various histopathological and immunohistochemical features of the primary, recurrent and metastatic tumours in evaluating their prognosis. The mean follow-up time was 5.5 years, ranging from 8 months to 17 years. Eight patients developed metastases and seven have died. The mean time from diagnosis to death due to metastasis was 3.8 years (range 1–6 years). The site of the primary tumour seemed to be most closely correlated to high metastatic risk. Only two of the sixteen limbal melanomas metastasised, whereas two of the four bulbar, all three tarsal and the only diffuse primary tumour caused metastatic disease. Two of the metastasising primary tumours were less than 1.5 mm thick, but all exceeded 0.8 mm in thickness. The mitotic rate, the amount of inflammatory infiltrate, the cell type or the presence of adjacent intraepithelial involvement did not obviously correlate to treatment outcome. Furthermore, the expression of S-100 protein and neuron-specific enolase (NSE), both suggested to be prognostic indicators in cutaneous melanoma, did not correlate to the tendency of the conjunctival melanomas to recur or metastasise.

Primary malignant melanoma of the conjunctiva is a rare neoplasm comprising less than 1% of malignant tumours of the eye (Grossniklaus *et al.*, 1987). In spite of many recent studies on the subject, reliable prognostic indicators to predict whether a particular tumour is likely to recur or metastasise have not been established, although thickness and location of the tumour, its mitotic rate or histopathological features of the associated acquired melanosis, if any, have been suggested as such (Jay, 1965; Silvers *et al.*, 1979; Crawford, 1980; Liesegang & Campbell, 1980; McGhee *et al.*, 1982; Benderitter *et al.*, 1985; Folberg *et al.*, 1985*b*; Guillen *et al.*, 1985; Jeffrey *et al.*, 1986; Stefani, 1986*a,b*).

S-100 protein, an acidic cytoplasmic and nuclear antigen of unknown function present in diverse normal and neoplastic human cell types (Kahn *et al.*, 1983; Vanstapel *et al.*, 1985, 1986; Haimoto *et al.*, 1987), and neuron-specific enolase (NSE), a rather widely distributed glycolytic isoenzyme primarily found in neuronal and neuroectodermal cells (Vinores *et al.*, 1984; Haimoto *et al.*, 1985), have been established as markers for melanoma of the skin (Cochran *et al.*, 1982; Nakajima *et al.*, 1982; Springall *et al.*, 1983; Kindblom *et al.*, 1984; Hachisuka *et al.*, 1986) and of the choroid (Nakajima *et al.*, 1982; Cochran *et al.*, 1983; Royds *et al.*, 1983; Margo & Lavellee, 1986; Garrido & Arra, 1987; Fuchs *et al.*, 1988). Both of these markers have also been put forward as possible prognostic indicators in cutaneous and choroidal melanoma (Royds *et al.*, 1982, 1983; Dhillon & Rode, 1982; Rode & Dhillon, 1984; Williams *et al.*, 1986; Hirano, 1986; Kernohan & Rankin, 1987).

The purpose of the present study was to evaluate whether any of the previously suggested histopathological features of conjunctival melanomas or their immunohistochemical reactivity for S-100 protein and NSE could be related to the prognosis in 26 patients with complete follow-up information.

### Materials and methods

### *Clinical review*

During the years 1967–1987 specimens from 26 white Caucasian patients with a primary malignant melanoma of the conjunctiva have been examined in the Ophthalmic Pathology Laboratory, Department of Ophthalmology, Helsinki University Central Hospital. The clinical histories, the treatment given and complete follow-up information (Figure 1) were retrieved from the records of all the hospitals where the patients had been treated for conjunctival melanoma and its metastases, from the Finnish Population and Cancer Registries and by contacting the patient when other sources of information were inconclusive. All death certificates and autopsy protocols were also reviewed. The location of conjunctival tumours (Table I) was recorded as limbal, caruncular, bulbar (without limbal or caruncular involvement), palpebral (limited to tarsal conjunctiva) or diffuse (involving two or more of these areas, or more than one quadrant of the bulbar conjunctiva). The size of the tumour (Table I) was either retrieved from the case records or estimated from clinical photographs by assuming that the average cornea is 11 mm in diameter.

### *Histological specimens*

In addition to the specimens on file in the Ophthalmic Pathology Laboratory, all available histopathological material from other hospitals where the patients had been treated was acquired for study. Both the original histological sections and the paraffin blocks for immunohistochemical studies could be retrieved from all but two of the 26 primary tumours, from nine of the eleven recurrent lesions, and from four of the five metastases that had been surgically removed or biopsied (Table I). Only slides were available from a fine needle aspiration biopsy of one lymph node metastasis (case 8/2). The depth of invasion was measured from the thickest part of the conjunctival tumours with a calibrated ocular micrometer. In seven cases a reliable measurement was not possible because the sections were either oblique or were not from the thickest part of the tumour as compared with the clinical photographs. In addition, the average number of mitoses per five high power (×400) microscopic fields was recorded as few (less than five mitoses), moderate (six to ten mitoses) or many (more than ten mitoses). The presence of proliferating intraepithelial melanocytes adjacent to the invasive tumour (primary acquired melanosis or radial growth phase) was recorded and classified according to Folberg *et al.* (1985*a*).

### *Immunohistochemical staining*

All specimens were formalin-fixed and paraffin-embedded. After deparaffinisation in xylene and rehydration in an ethanol series, endogenous peroxidase activity was destroyed by a 30-min treatment in methanol containing 0.5% hydrogen peroxide. The sections were then incubated for 30 min with normal serum (Vectastain ABC kits for Rabbit and

Correspondence: T. Kivelä.
Received 8 July 1988.
*Present address: Eye Clinic and Institute of Pathologic Anatomy, Karl-Marx-Universität, Liebigstraße 14 and 26, 7010 Leipzig, GDR.

**Figure 1** Clinical course of the 26 primary conjunctival melanomas studied. R = recurrent and M = metastatic tumour. Ec = local excision, en = enucleation, ex = exenteration, nd = radical neck dissection, ph = photocoagulation, cr = cryocoagulation, rd = radiotherapy and ch = chemotherapy. 1 = dead of metastatic melanoma, 2 = dead of intercurrent disease.

Mouse IgG; Vector Laboratories, Burlingame, CA, USA; dilution 1:50) in a moist chamber at room temperature, and for 60 min with the primary antibodies at 37°C. The primary rabbit antisera (anti-neuron specific enolase A589, lot 084; dilution 1:600 and anti-cow S100 Z311, lot 113; dilution 1:100) were purchased from Dakopatts a/s (Glostrup, Denmark). The monoclonal anti-macrophage antibody RPN.701 (lot 1; undiluted) recognising two determinants on human macrophages was obtained from Amersham Laboratories (Amersham, UK). It cross-reacts with some granulocytes and many squamous epithelia (Isaacson & Jones, 1983). DAKO-LC (lot 123; dilution 1:10), a mixture of two monoclonal antibodies recognising different epitopes on human leukocyte common antigen (Warnke *et al.*, 1983), a group of membrane glycoproteins on most types of human leukocytes, was also purchased from Dakopatts a/s.

Subsequently the sections were treated with biotinylated anti-rabbit and anti-mouse secondary antisera (Vectastain ABC kits; 1:200) and then with the avidin-biotinylated peroxidase complex (Vectastain ABC kit reagents A and B, both diluted 1:160) in a moist chamber at 37°C for 30 min. All immunoreagents were diluted with phosphate-buffered saline (PBS; pH 7.4) containing 2% (w/v) bovine serum albumin (E. Merck, Darmstadt, FRG). Between every step, the sections were washed for three 10-min changes in PBS.

The peroxidase reaction was developed with 3-amino-9-ethylcarbazole (Sigma; St Louis, MO; 40 mg dissolved in 12 ml of *N,N*-dimethylformamide and 200 ml of 0.05 M sodium acetate buffer, pH 5.0, containing 0.03% hydrogen peroxide) which gives a red reaction product contrasting with brown melanin pigments. To enable evaluation of the positive staining reaction in more heavily pigmented lesions the peroxidase reaction was also developed with 3,3′-diaminobenzidine tetrahydrochloride (Sigma; 150 mg in 16 ml of dimethylsulphoxide and 200 ml of PBS containing 0.03% hydrogen peroxide) which gives a dark brown reaction product resistant to hydrogen peroxide, melanin being then bleached for 18 h in a solution containing 3% (v/v) hydrogen peroxide and 1% (w/v) disodium hydrogen phosphate (Romeis, 1968).

The percentage of melanoma cells reacting positively for S-100 protein and neuron-specific enolase was recorded. The extent of lymphocytic infiltration (LC-positive cells) at the base of the tumour was graded as mild, moderate or strong according to Jeffrey *et al.* (1986). In addition, the number of infiltrating LC-positive lymphocytes and RPN.701-positive macrophages within the melanomas was graded as few (0–4 cells), moderate (5–14 cells) or many (more than 15 cells per ×400 microscopic field).

For comparative purposes, three cases of primary acquired melanosis of the conjunctiva with and three cases without melanocytic atypia (Folberg *et al.*, 1985*a*), as well as three conjunctival naevi, were selected from the files of the Ophthalmic Pathology Laboratory. In all specimens conjunctival nerve processes and Schwann cells served as internal positive controls for NSE and S-100 protein, respectively. Omission of the primary or secondary antibody and substitution of the primary antibody with normal rabbit serum or an unrelated murine monoclonal antibody (Anti-Cytokeratin PKK1; Labsystems, Helsinki, Finland) were used as negative controls.

**Table I** Patient characteristics and histopathological features of the conjunctival melanomas studied.[a]

| Case no. | Age (years) | Sex | Tumour type | Location | Size (mm) | Depth (mm) | Cell type | PAM[b] | Positive cells[c] S-100 | NSE | Lympho | Macro | Mitoses |
|---|---|---|---|---|---|---|---|---|---|---|---|---|---|
| 1 | 60 | F | Primary | Limbal | 3×5 | 0.6 | Epithelioid | bne | 95% | – | ++/+ | +++ | + |
| 2 | 48 | F | Primary | Limbal | 8×9 | 0.6 | Epithelioid | ins | 90% | – | +++/++ | + | + |
| 3 | 38 | F | Primary | Limbal | 3×5 | 0.6[d] | Epithelioid | pag | 75% | – | +++/++ | + | + |
| 4 | 75 | M | Primary | Limbal | 3×5 | 0.6[d] | Epithelioid | bhy | 90% | – | ++/++ | + | + |
| 5 | 70 | F | Primary | Limbal | 3×7 | 1.0[d] | Epithelioid | n/a | 95% | – | ++/+ | + | + |
| 6 | 82 | M | Primary | Limbal | 4×4 | 1.0[d] | Epithelioid | n/a | 90% | 35% | +/+ | ++ | ++ |
| 7/1 | 64 | M | Primary | Limbal | 5×6 | 1.0[d] | Mixed | n/a | 90% | 60% | +/+ | + | + |
| /2 | | | Recurrence | Limbal | 4×3 | 0.7[d] | Mixed | – | 95% | – | ++/+ | + | + |
| /3 | | | Recurrence | Limbal | 2×4 | 0.3 | Epithelioid | – | 95% | – | +/+++ | + | + |
| 8/1 | 73 | M | Primary | Limbal | 5×10 | 1.1[d] | Mixed | pag | 90% | – | ++/++ | +++ | + |
| /2 | | | Metastasis | Neck | n/a | n/a | Mixed | n/a | n/a | n/a | n/a | n/a | n/a |
| /3 | | | Recurrence | Bulbar | 1×2 | 0.2 | Epithelioid | – | 10% | – | +/+++ | + | + |
| 9 | 65 | M | Primary | Limbal | 4×4 | 1.5[d] | Pleomorphic | bhy | 80% | – | +++/+ | +++ | + |
| 10 | 63 | M | Primary | Limbal | 7×7 | 1.9[d] | Epithelioid | – | 85% | 80% | +/+ | | ++ |
| 11 | 42 | M | Primary | Limbal | 6×7 | 2.5 | Epithelioid | n/a | 50% | – | +++/+ | ++ | + |
| 12 | 53 | M | Primary | Limbal | 4×10 | 2.5[d] | Epithelioid | – | 85% | <1% | ++/+ | +++ | ++ |
| 13 | 69 | F | Primary | Limbal | 7×7 | 3.9[d] | Epithelioid | bhy | 85% | – | ++/++ | +++ | +++ |
| 14 | 67 | M | Primary | Limbal | 4×4 | n/a | Mixed | bne | 85% | – | +++/++ | ++ | + |
| 15 | 29 | M | Primary | Limbal | 4×6 | 2.2 | Epithelioid | – | 95% | 25% | +++/+++ | +++ | + |
| 16/1 | 66 | F | Primary | Limbal | 4×4 | n/a | Mixed | – | 95% | <1% | ++/+ | + | +++ |
| /2 | | | Recurrence | Limbal | n/a | 0.3 | Epithelioid | – | 80% | 50% | +/+ | + | + |
| 17/1 | 51 | F | Primary | Bulbar | 3×4 | 0.9 | Epithelioid | ine | n/a | n/a | n/a | n/a | + |
| /2 | | | Metastasis | Rib | n/a | n/a | Epithelioid | n/a | 85% | 70% | n/a | n/a | + |
| /3 | | | Recurrence | Palpebral | 7×12 | 2.9 | Epithelioid | n/a | 90% | 25% | +/+ | + | +++ |
| 18 | 49 | M | Primary | Bulbar | 4×9 | 2.2 | Epithelioid | – | 95% | 1% | ++/++ | ++ | + |
| 19/1 | 43 | M | Primary | Bulbar | 7×8 | 2.3[d] | Epithelioid | – | 80% | 25% | +/+ | +++ | ++ |
| /2 | | | Metastasis | Parotis | 20×20 | n/a | Epithelioid | n/a | 50% | 50% | n/a | n/a | +++ |
| /3 | | | Metastasis | Neck | n/a | n/a | Epithelioid | n/a | 20% | – | +++/++ | +++ | n/a |
| /4 | | | Metastasis | Neck | n/a | n/a | Epithelioid | n/a | n/a | n/a | n/a | n/a | n/a |
| 20 | 69 | F | Primary | Bulbar | 5×6 | 3.9 | Epithelioid | ins | 60% | <1% | ++/++ | + | +++ |
| 21 | 81 | M | Primary | Caruncle | 6×7 | 3.6 | Spindle | bne | 75% | – | ++/+ | + | + |
| 22/1 | 29 | M | Primary | Caruncle | 3×5 | n/a | Epithelioid | ins | 95% | – | ++/+ | + | + |
| /2 | | | Recurrence | Bulbar | 3×4 | 2.7 | Epithelioid | – | 95% | – | +/+ | + | ++ |
| 23 | 74 | F | Primary | Palpebral | n/a | 2.5[d] | Epithelioid | n/a | 60% | – | ++/++ | + | ++ |
| 24/1 | 57 | F | Primary | Palpebral | 6×12 | n/a | Mixed | n/a | 90% | – | +/++ | + | n/a |
| /2 | | | Recurrence | Diffuse | n/a | n/a | Mixed | ins | 90% | – | +++/+ | + | ++ |
| /3 | | | Recurrence | Diffuse | n/a | 0.7 | Epithelioid | ins | 95% | – | +++/+++ | ++ | + |
| /4 | | | Metastasis | Neck | 16×16 | n/a | Mixed | n/a | 65% | <1% | +/+ | + | ++ |
| 25/1 | 85 | M | Primary | Palpebral | n/a | n/a | Epithelioid | n/a | n/a | n/a | n/a | n/a | n/a |
| /2 | | | Recurrence | Palpebral | n/a | n/a | Epithelioid | n/a | n/a | n/a | n/a | n/a | n/a |
| /3 | | | Recurrence | Orbital | n/a | n/a | Epithelioid | n/a | n/a | n/a | n/a | n/a | n/a |
| /4 | | | Recurrence | Orbital | 12×16 | n/a | Epithelioid | n/a | 50% | 15% | +/+ | + | +++ |
| 26/1 | 47 | M | Primary | Diffuse | 13×18 | 8.8 | Mixed | – | 80% | 1% | ++/+ | + | + |
| /2 | | | Metastasis | Skin | 30×30 | n/a | Epithelioid | n/a | 35% | – | +/+ | + | +++ |

[a]n/a = data not available or applicable, + = few, ++ = moderate, +++ = many; [b]Predominant type of associated primary acquired melanosis (PAM): bhy = basilar hyperplasia, bne = basilar nests, ine = intraepithelial nests, pag = pagetoid involvement, ins = *in situ*-like involvement, and – = not present (Folberg *et al.*, 1985*a*); [c]Percentage of melanoma cells positive for S-100 protein and neuron-specific enolase (NSE), number of LC-positive lymphocytes around/within the tumour and number of RPN.701-positive macrophages within the tumour; [d]Extends to the deep margin of the specimen.

### Results

### Clinical course

The mean follow-up time in the present series was 5.5 years, ranging from 8 months to 17 years (Figure 1). The primary therapy most often used was local resection, sometimes combined with cryocoagulation or radiotherapy. Enucleation, exenteration and radical neck dissection were seldom used (Figure 1). Eight of the 26 primary conjunctival melanomas studied have later recurred and eight have metastasised (Table II). Seven of the eight metastatic cases have resulted in the death of the patient (Figure 1). Among the 16 primary limbal melanomas, recurrence was observed three times and metastasis twice. The latter were large tumours which extended to the deep margin of the histopathological specimen (Table I). Furthermore, nine patients with limbal melanomas have survived 5 years or longer without either recurrence or metastasis. In contrast, six of the ten patients with a bulbar, caruncular, palpebral or diffuse primary melanoma have died of metastatic disease 2–6 years after the initial operation (mean interval 3.8 years; Figure 1). Of the remaining four patients, three are alive but have not yet been followed for 5 years and one patient with a large caruncular tumour died of intercurrent disease soon after the operation.

### Light microscopy

In four cases, all of which were limbal and have neither recurred nor metastasised, the thickness of the primary tumour was 0.8 mm or less (Table I). Two of the six primary tumours that later metastasised, and whose depth could reliably be determined, were less than 1.5 mm thick. Only four of the 12 primary melanomas 1.5 mm or more in depth have metastasised. The primary tumour was the thickest lesion diagnosed before metastases became apparent in every case.

Nineteen primary tumours were of the epitheloid cell type, six of the mixed cell type and one of the spindle cell type (Table I). One of the epithelioid cell melanomas was very pleomorphic and contained many giant tumour cells. Five epithelioid and three mixed cell melanomas metastasised. Adjacent intraepithelial involvement by atypical melanocytes (primary acquired melanosis or radial growth phase) was observed in 12 of the 19 cases of primary melanoma where sufficient surrounding epithelium was available for study (Table I). Three melanomas with and two without intraepithelial involvement later metastasised. The mitotic rate was low in most of the primary tumours and it was generally not noticeably higher in cases that later recurred or metastasised, although in several secondary tumours the number of mitoses was greater than in the corresponding primaries (Table I). Frank invasion of blood or lymphatic vessels was observed in one case only (case 19).

### Immunohistochemistry

Tumour cells reacting with the antiserum to S-100 protein (Figure 2b) were found in all conjuctival melanomas studied (Table I). The intensity of the reaction and the number of positive cells varied between individual tumours, and pigmented cells were generally only faintly labelled. The deeper parts of the tumour could be labelled more intensely than their superficial parts, but in most cases there was no obvious difference in this respect. These deeper parts generally consisted of smaller cells without marked nuclear pleomorphism and lacked prominent nucleoli. Nests of naevoid cells were noted in several specimens adjacent to the melanoma, and these were often more strongly labelled than the melanoma cells. The overall number of positive melanoma cells tended to be smaller in metastatic lesions than in corresponding primary tumours (Table I). The atypical melanocytes in primary acquired melanosis reacted in every case strongly for S-100 protein, rendering evaluation of its extent easy (Figure 2g and h). Many normal melanocytes of the conjunctiva were also moderately labelled (Figure 2f), especially in the palpebral conjunctiva. The three conjunctival naevi were uniformly positive for S-100 protein.

Ten of the 26 primary melanomas studied reacted positively for neuron-specific enolase (Figure 2c). More than 20% of the neoplastic cell population was positively labelled in five cases only, one of which has metastasised (Table I). In two of the four metastatic lesions available for study at least half of the tumour cells were labelled, while the other two remained essentially negative. Normal melanocytes of the conjunctiva, as well as atypical melanocytes in primary acquired melanosis and conjunctival naevi were negative for NSE without exception.

The amount of the inflammatory infiltrate was generally greater around the tumour than it was within it (Table I). Larger numbers of LC-positive lymphocytes and macrophages reacting with the anti-macrophage antibody were observed within the primary tumour in eight and seven cases, respectively (Figure 2d and e). The infiltrating lymphocytes were often situated in the immediate vicinity of blood vessels, while macrophages were more randomly distributed. The antimacrophage antibody additionally reacted with the conjunctival epithelial cells in a way similar to the anti-keratin antibody. The amounts of lymphocytes and macrophages showed no obvious correlation to treatment outcome (Table I).

**Table II** Clinical characteristics of the 26 primary conjunctival melanomas according to their location

| *Location* | *Number* | *Mean age (years)* | *Recurrences* | *Metastases* |
|---|---|---|---|---|
| Limbal | 16 (62%) | 60 | 3 (19%) | 2 (12%) |
| Bulbar | 4 (15%) | 53 | 1 (25%) | 2 (50%) |
| Caruncular | 2 (8%) | 55 | 1 (50%) | – |
| Palpebral | 3 (12%) | 72 | 3 (100%) | 3 (100%) |
| Diffuse | 1 (4%) | 47 | – | 1 (100%) |
| Total | 26 | 59 | 8 (31%) | 8 (31%) |

### Discussion

In line with cutaneous melanomas, it has been documented that the depth of invasion, either alone (Silvers *et al.*, 1979; Liesegang & Campbell, 1980; Jeffrey *et al.*, 1986; Folberg *et al.*, 1985*b*; McGhee *et al.*, 1982) or in combination with the rate of mitosis (Stefani, 1986*a,b*), is an important prognostic factor of conjunctival melanomas. However, care should be taken to section all specimens perpendicularly to the surface of the conjunctiva at several levels to ensure reliable results (Silvers *et al.*, 1979; Folberg *et al.*, 1985*b*). Since this is not always possible in retrospective studies, the critical thickness indicative of high metastatic risk remains controversial. Although initially reported to be 1.5 mm (Silvers *et al.*, 1979; McGhee *et al.*, 1982; Jeffrey *et al.*, 1986), a limit of 0.8 mm has more recently been put forward (Folberg *et al.*, 1985*b*). There were two primary tumours leading to metastatic disease that were less than 1.5 mm in thickness in the present material, and even more superficially invasive conjunctival melanomas are known to metastasise, supporting the latter limit (Crawford, 1980; Folberg *et al.*, 1985*b*). The value of the depth of invasion as a prognostic indicator is, however, diminished by the fact that many tumours more than 1.5 mm in depth do not metastasise, as was also observed in the present study.

Other clinicopathological features indicative of poor prognosis have been suggested. These include origin from previously existing naevus (Liesegang & Campbell, 1980), young age of the patient (Crawford, 1980), involvement of the caruncular or palpebral conjunctiva (Jay, 1965; Jeffrey *et al.*, 1986; Crawford, 1980; Silvers *et al.*, 1979; Stefani, 1986*a,b*), high rate of mitosis (Crawford, 1980; Folberg *et al.*, 1985*b*;

**Figure 2** (**a**) Epithelioid cell type primary malignant melanoma of the conjunctiva with marked inflammatory infiltration among tumour nests (Case 15; HE, ×350). (**b**) The neoplastic cells are strongly positive for S-100 protein. Inflammatory cells remain negative (×350). (**c**) Some of the melanoma cells show weak to moderate positive reaction for neuron-specific enolase (×350). (**d**) Most inflammatory cells are positive for leukocyte common antigen, while melanoma cells remain negative (×350). (**e**) Many of the inflammatory cells react with the anti-macrophage antibody (×350). (**f**) Weak reaction for S-100 protein in normal melanocytes of the conjunctiva (arrowheads). The dendritic suprabasal cell (arrow) is probably a Langerhans cell (×270). (**g**) Infiltration of conjunctival epithelium by abnormal melanocytes in primary acquired melanosis (HE, ×270). (**h**) These melanocytes react strongly for S-100 protein (×270).

Benderitter *et al.*, 1985; Stefani, 1986*a,b*), weak inflammatory reaction (Crawford, 1980; Folberg *et al.*, 1985*b*), invasion of blood vessels (McGhee *et al.*, 1982; Benderitter *et al.*, 1985), and atypia observed in the associated primary acquired melanosis (PAM) (Folberg *et al.*, 1985*b*). In the present series, the amount of lymphocytes or macrophages demonstrable immunohistochemically, the cell type or the presence of primary acquired melanosis were not obviously correlated to prognosis. The mitotic rate, although often greater in metastatic and recurrent lesions, was not particularly high in most of the corresponding primary tumours. As in previous series, involvement of palpebral conjunctiva was associated with poor prognosis, which may in part be dependent on the greater size of the primary tumours (Silvers *et al.*, 1979; Crawford, 1980; Jeffrey *et al.*, 1986). Neither of the two caruncular cases in the present series allow any firm conclusions as to treatment outcome, but caruncular melanomas are thought to have a rather poor prognosis (Silvers *et al.*, 1979; Crawford, 1980). It is, indeed, quite clear that limbal melanomas have a much better prognosis than their counterparts in other areas of the conjunctiva.

S-100 protein was detected in all conjunctival melanomas and naevi studied, confirming previous observations

(Kindblom et al., 1984; Holbach & Hofmann, 1987). As has been reported for cutaneous and choroidal melanomas, the reaction intensity in individual cells was quite variable and deeply pigmented cells were generally only weakly reactive (Nakajima et al., 1982; Royds et al., 1982; Springall et al., 1983; Hachisuka et al., 1986; Fuchs et al., 1988). Whether immunoreactivity for S-100 protein is a prognostic indicator in cutaneous melanomas is disputed. While some authors have failed to observe any correlation to prognosis (Hachisuka et al., 1986), others have claimed that strong reaction for S-100 protein in primary melanomas could denote either improved survival (Kernohan & Rankin, 1987) or early metastasis (Rode & Dhillon, 1984; Williams et al., 1986). In the present series, there was no difference in the reactivity between the primary lesions that had later metastasised and those that had not. However, the number of positively staining tumour cells was smaller and the overall reaction intensity generally less intense in metastatic than in primary lesions. Such a difference has not been reported for cutaneous melanomas (Nakajima et al., 1982; Springall et al., 1983; Hachisuka et al., 1986).

The antiserum to neuron-specific enolase (NSE) reacted only with a minority of the melanoma cells and did not label any of the naevi studied. These results are in line with several previous reports on cutaneous melanoma, in which a weak and patchy reaction for NSE has been obtained in less than one half of the studied tumours (Springall et al., 1983; Hachisuka et al., 1986; Thomas et al., 1987). In other studies, however, almost all cutaneous melanomas have been positively labelled (Dhillon & Rode, 1982; Rode & Dhillon, 1984; Royds et al., 1982; Hirano, 1986), possibly indicating differences in antibody specificity.

Rode & Dhillon (1984) have suggested that NSE is preferentially expressed in metabolically active malignant melanocytes and have shown that primary tumours strongly positive for NSE give rise to metastases more rapidly than those reacting only weakly. Some metastases and recurrences of the conjunctival melanomas presently studied did in fact have greater numbers of NSE-positive tumour cells than the corresponding primary lesions, which would be compatible with this theory. However, at least five of the eight primary conjunctival melanomas that later metastasised contained insignificant numbers of NSE-positive tumour cells, and three of the five cases in which more than one-fifth of the tumour cells were positive for NSE neither recurred nor metastasised, indicating that immunoreactivity for NSE cannot predict which lesions are prone to metastasis. Indeed, even in the study by Rode & Dhillon (1984), weakly NSE-positive primary cutaneous melanomas metastasised more often than strongly positive tumours, and several metastases showed a weaker reaction than the corresponding primary tumours. Furthermore, none of the six NSE-positive skin melanomas of Springall et al. (1983) metastasised, and none of their 25 metastatic melanomas were positive for NSE.

Although our results do not support the theory, suggested for skin melanomas, that immunoreactivity for S-100 protein and NSE could be used as prognostic indicators in conjunctival melanoma, S-100 protein was found to be a useful adjunct not only in the diagnosis of this disease, but also in easily delineating the extent of the associated intraepithelial involvement. Since a number of recent histopathological studies on conjunctival melanomas have shown that conventional histopathological criteria do not provide consistent criteria for predicting their prognosis, attention should be turned towards new metabolic and antigenic properties of these tumours, as well as to those host factors that ultimately determine whether micrometastases will lead to frank metastatic disease.

The technical assistance of Mrs Marjatta Koikkalainen and Mrs Pirkko Yliharju is gratefully acknowledged. We wish to thank the Finnish Cancer Registry, the Finnish Population Registry, and all other institutes for their invaluable help in making the follow-up information and histopathological material available for the present study. This study was in part supported by the Finnish Cancer Foundation

### References

BENDERITTER, T., BERARD, M., BONERANDI, J.J. & LEBREUIL, G. (1985). Les proliferations melaniques de la conjonctive. Etude histopathologique a propos d'une serie de 40 cas. *J. Fr. Ophtalmol.*, **8**, 411.

COCHRAN, A.J., WEN, D.-R., HERSCHMAN, H.R. & GAYNOR, R.B. (1982). Detection of S-100 protein as an aid to the identification of melanocytic tumors. *Int. J. Cancer*, **30**, 295.

COCHRAN, A.J., HOLLAND, G.N., WEN, D.-R. & 4 others (1983). Detection of cytoplasmic S-100 protein in primary and metastatic intraocular melanomas. *Invest. Ophthalmol. Vis. Sci.*, **24**, 1153.

CRAWFORD, J.B. (1980). Conjunctival melanomas: prognostic factors. A review and an analysis of a series. *Trans. Am. Ophthalmol. Soc.*, **78**, 467.

DHILLON, A.P. & RODE, J. (1982). Patterns of staining for neurone specific enolase in benign and malignant melanocytic lesions of the skin. *Diagn. Histopathol.*, **5**, 169.

FOLBERG, R., McLEAN, I.W. & ZIMMERMAN, L.E. (1985*a*). Primary acquired melanosis of the conjunctiva. *Human Pathol.*, **16**, 129.

FOLBERG, R., McLEAN, I.W. & ZIMMERMAN, L.E. (1985*b*). Malignant melanoma of the conjunctiva. *Human Pathol.*, **16**, 136.

FUCHS, U., KIVELÄ, T., TARKKANEN, A. & LAATIKAINEN, L. (1988). Histopathology of enucleated intraocular melanomas irradiated with cobalt and ruthenium plaques. *Acta Ophthalmol.*, **66**, 255.

GARRIDO, C.M. & ARRA, A. (1987). Value of S100 protein in the study of nevus and ocular melanomas. *Ophthalmologica*, **194**, 201.

GROSSNIKLAUS, H.E., GREEN, W.R., LUCKENBACH, M. & CHAN, C.C. (1987). Conjunctival lesions in adults. A clinical and histopathological review. *Cornea*, **6**, 78.

GUILLEN, F.J., ALBERT, D.M. & MIHM, M.C. JR. (1985). Pigmented melanocytic lesions of the conjunctiva – a new approach to their classification. *Pathology*, **17**, 275.

HACHISUKA, H., SAKAMOTO, F., NOMURA, H., MORI, O. & SASAI, Y. (1986). Immunohistochemical study of S-100 protein and neuron specific enolase (NSE) in melanocytes and the related tumors. *Acta Histochem.*, **80**, 215.

HAIMOTO, H., HOSODA, S. & KATO, K. (1987). Differential distribution of immunoreactive S100α and S100β-proteins in normal nonnervous human tissues. *Lab. Invest.*, **57**, 489.

HAIMOTO, H., TAKAHASHI, Y., KOSHIKAWA, T., NAGURA, H. & KATO, K. (1985). Immunohistochemical localization of γ-enolase in normal human tissues other than nervous and neuroendocrine tissues. *Lab. Invest.*, **52**, 257.

HIRANO, T. (1986). Immunohistochemical study of malignant melanoma. *Acta Pathol. Jpn.*, **36**, 733.

HOLBACH, L. & HOFMANN, C. (1987). S-100 Protein in nichtpigmentierten melanozytären und neuroektodermalen Tumoren der Konjunktiva. *Klin. Monatsbl. Augenheilkd.*, **190**, 105.

ISAACSON, P.G. & JONES, D.B. (1983). Immunohistochemical differentiation between histiocytic and lymphoid neoplasms. *Histochem. J.*, **15**, 621.

JAY, B. (1965). Naevi and melanomata of the conjunctiva. *Br. J. Ophthalmol.*, **49**, 169.

JEFFREY, I.J.M., LUCAS, D.R., McEWAN, C. & LEE, W.R. (1986). Malignant melanoma of the conjunctiva. *Histopathology*, **10**, 363.

KAHN, H.J., MARKS, A., THOM, H. & BAUMAL, R. (1983). Role of antibody to S100 protein in diagnostic pathology. *Am. J. Clin. Pathol.*, **79**, 341.

KERNOHAN, N.M. & RANKIN, R. (1987). S-100 protein: a prognostic indicator in cutaneous malignant melanoma? *Histopathology*, **11**, 1285.

KINDBLOM, L.-G., LODDING, P., ROSENGREN, L., BAUDIER, J. & HAGLID, K. (1984). S-100 protein in melanocytic tumours. An immunohistochemical investigation of benign and malignant melanocytic tumours and metastases of malignant melanoma and a characterization of the antigen in comparison to human brain. *Acta Pathol. Microbiol. Immunol. Scand.* (*A*), **92,** 219.

LIESEGANG, T.J. & CAMPBELL, R.J. (1980). Mayo clinic experience with conjunctival melanomas. *Arch. Ophthalmol.*, **98,** 1385.

McGHEE, C.N.J., NI, C., ALBERT, D.M. & CHU, F.R. (1982). Conjunctival melanoma. *Int. Ophthalmol. Clin.*, **22,** 35.

MARGO, C.E. & LAVELLEE, M. (1986). Gamma-enolase activity in choroidal melanoma. *Graefe's Arch. Clin. Exp. Ophthalmol.*, **224,** 374.

NAKAJIMA, T., WATANABE, S., SATO, Y., KAMEYA, T., SHIMOSATO, Y. & ISHIHARA, K. (1982). Immunohistochemical demonstration of S100 protein in malignant melanoma and pigmented nevus, and its diagnostic application. *Cancer,* **50,** 912.

ROMEIS, B. (1968). *Mikroskopische Technik,* 16th edition, p. 281. R. Oldenbourg Verlag: Munich.

RODE, J. & DHILLON, A.P. (1984). Neurone specific enolase and S100 protein as possible prognostic indicators in melanoma. *Histopathology,* **8,** 1041.

ROYDS, J.A., PARSONS, M.A., RENNIE, I.G., TIMPERLEY, W.R. & TAYLOR, C.B. (1982). Enolase isoenzymes in benign and malignant melanocytic lesions. *Diagn. Histopathol.*, **5,** 175.

ROYDS, J.A., RENNIE, I.G., PARSONS, M.A., TIMPERLEY, W.R. & TAYLOR, C.B. (1983). Enolase isoenzymes in uveal melanomas – a possible parameter of malignancy. *Br. J. Ophthalmol.*, **67,** 244.

SILVERS, D.N., JAKOBIEC, F.A., FREEMAN, T.R., LEFKOWITCH, J.H. & ELIE, R.C. (1979). Melanoma of the conjunctiva: a clinicopathologic study. In *Ocular and Adnexal Tumors,* Jakobiec, F.A. (ed) p. 583. Aesculapius: Alabama.

SPRINGALL, D.R., GU, J., COCCHIA, D. & 6 others (1983). The value of S-100 immunostaining as a diagnostic tool in human malignant melanomas. A comparative study using S-100 and neuron-specific enolase antibodies. *Virchows Arch.* (*Pathol. Anat.*), **400,** 331.

STEFANI, F.H. (1986*a*). Das maligne Melanom der Bindehaut – Klinischer Verlauf und histopathologischer Befund. *Fortschr. Ophthalmol.*, **83,** 141.

STEFANI, F.H. (1986*b*). A prognostic index for patients with malignant melanoma of the conjunctiva. *Graefe's Arch. Clin. Exp. Ophthalmol.*, **224,** 580.

THOMAS, P., BATTIFORA, H., MANDERINO, G.L. & PATRICK, J. (1987). A monoclonal antibody against neuron-specific enolase. Immunohistochemical comparison with a polyclonal antiserum. *Am. J. Clin. Pathol.*, **88,** 146.

VANSTAPEL, M.-J., PEETERS, B., CORDELL, J. & 4 others (1985). Production of monoclonal antibodies directed against antigenic determinants common to the α- and β-chain of bovine brain S-100 protein. *Lab. Invest.*, **52,** 232.

VANSTAPEL, M.-J., GATTER, K.C., DE WOLF-PEETERS, C., MASON, D.Y. & DESMET, V.D. (1986). New sites of human S-100 immunoreactivity detected with monoclonal antibodies. *Am. J. Clin. Pathol.*, **85,** 160.

VINORES, S.A., BONNIN, J.M., RUBINSTEIN, L.J. & MARANGOS, P.J. (1984). Immunohistochemical demonstration of neuron-specific enolase in neoplasms of the CNS and other tissues. *Arch. Pathol. Lab. Med.*, **108,** 536.

WARNKE, R.A., GATTER, K.C., FALINI, B. & 7 others (1983). Diagnosis of human lymphoma with monoclonal antileukocyte antibodies. *N. Engl. J. Med.*, **309,** 1275.

WILLIAMS, R.A., RODE, J., DHILLON, A.P., JARVIS, L.R., SKINNER, J.M. & JAMAL, O. (1986). Measuring S100 protein and neurone specific enolase in melanocytic tumours using video image analysis. *J. Clin. Pathol.*, **39,** 1096.